# Clinical utility of thresholds versus CRD

**DOI:** 10.1186/2045-7022-1-S1-S29

**Published:** 2011-08-12

**Authors:** Carsten Bindslev-Jensen

**Affiliations:** 1 Odense University Hospital, Department of Dermatology and Allergy Center, Odense, Denmark

## 

Since food challenge is time consuming and not always without a risk for the patient, surrogate parameters have been introduced. Among the best studied are case history, size of Skin Prick Test and the level of specific IgE towards a food allergen. In the later case, decision points (ie. the level of specific IgE above which there would be a 95 % probability of the patient being challenge positive) have been introduced for various foods, including, egg, milk, nuts and peanuts. Two major problems arise, however, from such an approach. Firstly, the decision point may vary considerably between centres. This has been shown for hen’s egg, where different centres have published decision points varying from >0,35 to above 14 kIU/l. Secondly, in many cases, cross reacting antibodies may limit the validity of the decision point. An example is seen in peanut allergy, where a level above 10 kIU/l is considered positive in most published papers; but higher levels of peanut IgE are often detected in pollen allergic patients with high levels of IgE towards grass and birch. Most foods contain several allergenic proteins, with varying clinical relevance. Peanut contain several allergens, of which some are clinically irrelevant but important due to cross reaction with IgE against pollen whereas others, especially the protein ara H2, are directly correlated the clinical disease. Component resolved diagnostics (CRD) may thus present a major step forward in the search for surrogate parameters. The ideal surrogate parameter should be able to discriminate between positive and negative challenge and also to correlate to disease severity and clinical sensitivity (threshold). Threshold is an important parameter to establish both in the single patient, facilitating tailor made guidelines for the patient, and in the community. Measurement of specific to CRD’s would hopefully result in a better correlation to threshold than conventional techniques, but this idea remains yet to be proven. In the peanut example above, although a very nice decision point for ara H2 was established, less convincing correlations to clinical threshold in the patient population was found.

